# Effect of tiotropium inhaler use on mortality in patients with tuberculous destroyed lung: based on linkage between hospital and nationwide health insurance claims data in South Korea

**DOI:** 10.1186/s12931-019-1055-5

**Published:** 2019-05-06

**Authors:** Ho Cheol Kim, Tae Hoon Kim, Ye-Jee Kim, Chin Kook Rhee, Yeon-Mok Oh

**Affiliations:** 1 Department of Pulmonary and Critical Care Medicine, Asan Medical Center, University of Ulsan College of Medicine, Seoul, South Korea; 2 Department of Internal Medicine, CHA Bundang Medical Center, CHA University, Seongnam, South Korea; 3 Department of Clinical Epidemiology and Biostatistics, Asan Medical Center, Seoul, South Korea; 4 Division of Pulmonary, Allergy and Critical Care Medicine, Department of Internal Medicine, Seoul St. Mary’s Hospital, College of Medicine, The Catholic University of Korea, Seoul, South Korea

**Keywords:** Tuberculosis, Tiotropium, Propensity score, Mortality

## Abstract

**Background:**

Although bronchodilator inhaler therapy can improve lung function in patients with tuberculous destroyed lung (TDL), its effect on mortality has not been studied. We evaluated the effect of tiotropium inhaler therapy on mortality in patients with TDL.

**Methods:**

A retrospective cohort of 963 patients with TDL was followed for up to ten years by linking hospital and nationwide health insurance claims data. We compared patients receiving tiotropium inhaler with patients without tiotropium after matching with propensity scores. In addition, we elucidated the risk factors of mortality using Cox proportional hazards model.

**Results:**

After the propensity score matching, the baseline characteristics were balanced in both the tiotropium group (*n* = 105) and the non-tiotropium group (n = 105); including mean age (63.9 vs. 64.4 years, *P* = 0.715), mean forced expiratory volume in 1 s (FEV_1_) (45.0 vs. 45.3%, *P* = 0.903), and others. After the propensity score matching, the tiotropium group showed better survival than the non-tiotropium group (median survival period: not reached for the tiotropium group vs. 7.24 years for the non-tiotropium group, Prentice-Wilcoxon test, *P* = 0.008). Multivariate Cox proportional hazard analysis revealed that tiotropium inhaler usage was associated with lower risk of mortality in the multivariate analysis (HR, 0.560; 95% CI, 0.380–0.824; *P* = 0.003) after adjusting age, sex, BMI, smoking history, mMRC dyspnea score, Charlson Comorbidity Index, concomitant COPD diagnosis, FEV_1_, X-ray severity score, and home oxygen usage.

**Conclusions:**

Our results suggest that tiotropium inhaler is associated with decreased all-cause mortality in TDL. Further prospective study is required for validation.

**Electronic supplementary material:**

The online version of this article (10.1186/s12931-019-1055-5) contains supplementary material, which is available to authorized users.

## Introduction

Previous tuberculosis (TB) infection can cause extensive destruction of the lung parenchyma, resulting in tuberculous destroyed lung (TDL) [1]. TDL is of particular concern in South Korea, a country with an intermediate TB burden as it can lead to respiratory failure requiring mechanical ventilation [2, 3]. In addition, prior TB infection has been found to cause chronic airflow obstruction, even after adjustment for smoking [4, 5]. Patients with TDL and airflow limitation can have higher frequencies of acute exacerbation, which might affect the clinical course of the disease [6]. Although the optimal treatment for patients with TDL has not been well investigated, inhaler therapy is often used, especially in patients with airflow obstruction [7]. Kim et al. found that treatment with indacaterol significantly improved trough forced expiratory volume in 1 s (FEV_1_) compared to placebo (treatment differences: 0.14 L) after 8 weeks in patients with TDL and moderate-to-severe airflow limitation [8]. However, no studies have evaluated the role of inhaler therapy in the mortality of patients with TDL.

In South Korea, the National Health Insurance Service is a universal health coverage system that provides care for nearly all of the population. Since national insurance claims data includes healthcare utilization information for both inpatient and outpatient services and provides patient demographics, diagnosis, and prescribed medication, this information is broader and potentially more accurate than individual hospital data [9]. Thus, the current study aimed to evaluate the effect of tiotropium on mortality in patients with TDL through the linkage of hospital data with nationwide health insurance claims data.

## Material and methods

### Study populations

**Fig. 1:**
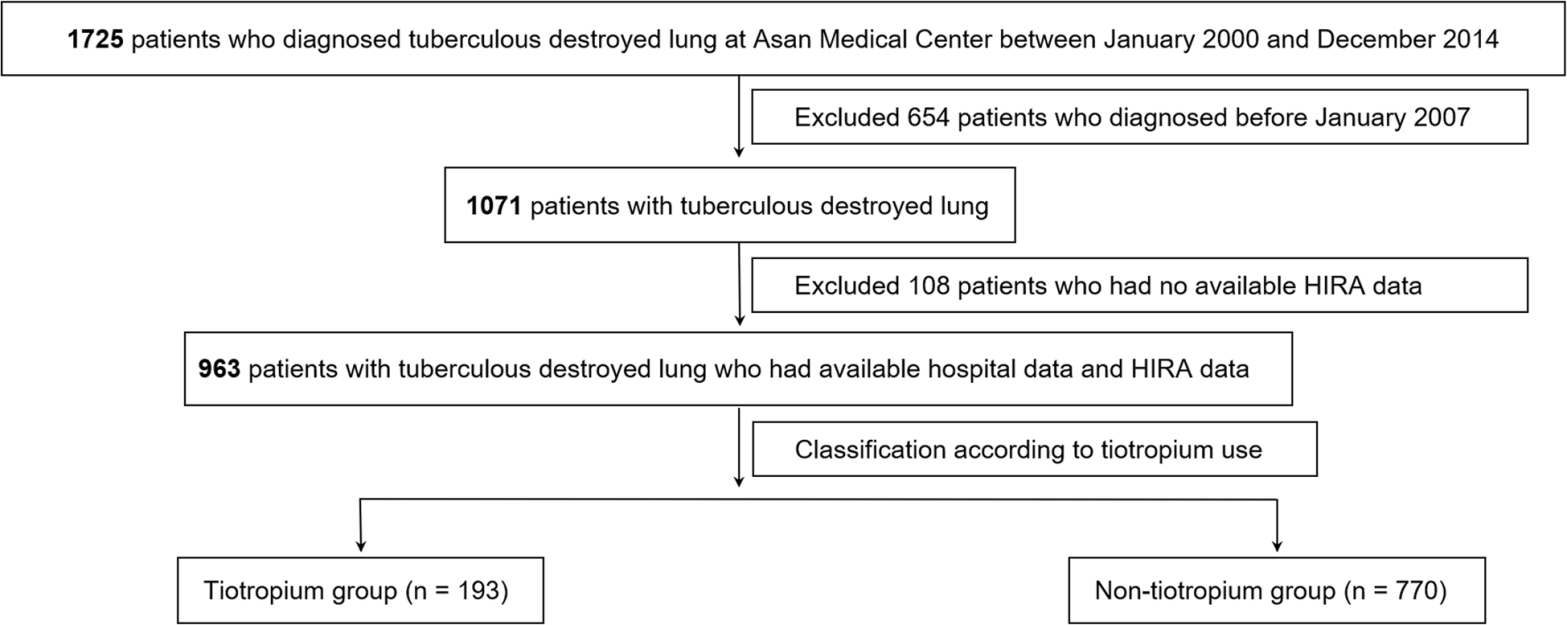
Study flow chart

We identified a total of 1071 patients diagnosed with TDL from January 2007 to December 2014 from the electronic medical records at Asan Medical Center, a tertiary referral hospital in South Korea. TDL was defined as parenchymal damage on chest radiograph due to sequelae from previous TB infection. Among these patients, a total of 963 patients (89.9%) with available data in nationwide health insurance claims database were enrolled in the study. Subsequently, we classified study patients into two groups, tiotropium group, which included patients who had been prescribed the tiotropium inhaler for ≥360 days during the total follow-up period, and the non-tiotropium group, defined as patients who never, or for < 360 days, had been prescribed tiotropium inhaler during the follow-up period. All eligible patients were followed up for at least 360 days and up to 10 years. The study flow chart is shown in Fig. 1. The study protocol was approved by the Institutional Review Board of Asan Medical Center (2016–1028), and the requirement for informed consent was waived due to the retrospective nature of the study.

### Data collection

Initially, data regarding patient age, sex, body mass index (BMI), smoking history, modified Medical Research Council (mMRC) score [10], comorbidity measured by the Charlson Comorbidity Index (CCI) [11], pulmonary function, prescription of tiotropium or inhaled corticosteroids/long-acting beta-2 agonist (ICS/LABA) inhaler history, and home oxygen usage were collected from electronic medical records in Asan Medical Center. At the time of data acquisition, only fluticasone/salmeterol and budesonide/formoterol were available ICS/LABA in South Korea. Spirometry and diffusing capacity of the lung for carbon monoxide (DLco) were measured according to recommendations and the results are expressed as percentages of the normal predicted values [12, 13]. To evaluate the severity of TDL of included patients, we evaluated posteroanterior chest radiographs of all patients and semi-quantified damage of the upper, middle, and lower regions of right and left lungs with a total severity score of 0 to 6 as previously described [7]. Then, hospital medical records of study population were linked to the Health Insurance Review and Assessment Service (HIRA) database which contains information on insurance claims for reimbursements from all medical institutions in Korea. This process was carried out via billing statement identification code after de-identification to protect personal data. The HIRA database includes general demographic data, diagnosis codes based on the 10th revision of the International Statistical Classification of Diseases and Related Health Problems (ICD-10), type of medical institution where diagnosis and/or treatment were made, medications prescribed, and medical costs. For outpatient visits, only visits due to a respiratory cause (tuberculosis [ICD-10 codes: A160x, A162, B90, and B909], pulmonary embolism [ICD-10 codes: I26, I260, and I269], chronic obstructive pulmonary disease [ICD-10 codes: J449, J42–44], pneumonia [ICD-10 codes: J12–17], adult respiratory distress syndrome [ICD-10 code: J80], dyspnea [ICD-10 code: R060], and other disorders of lung [ICD-10 code: J984]) were used for the analysis. In addition to hospital electronic medical records, clinical follow-up data, including patient mortality, were obtained from the HIRA database through December 2016.

### Statistical analysis

Data from the tiotropium and non-tiotropium groups were compared using Student’s t-test or the Mann-Whitney U test (continuous variables) and the chi-squared test or Fisher’s exact test (categorical data). All *P*-values were two-tailed, with statistical significance set at *P* <  0.05. To avoid bias from the retrospective design of this cohort study, we performed propensity score matching to reduce potential confounding by non-random assignment or unbalanced covariates between the tiotropium and non-tiotropium groups. Propensity scores were calculated using logistic regression analysis with the following covariates: age, sex, BMI, smoking history, mMRC dyspnea score, Charlson Comorbidity Index, concomitant asthma and chronic obstructive pulmonary disease (COPD) diagnosis, pulmonary function test (FEV_1_), X-ray severity score, and home oxygen usage. Model discrimination was assessed with C statistics (0.896), and model calibration was assessed with Hosmer-Lemeshow statistics (χ2 = 4.879; df = 8; *P* = 0.770). After calculating the predicted probabilities, we matched each tiotropium user to one non-user using the Greedy 5-to-1 digit-matching algorithm [14]. Balances in the distribution of baseline covariates were estimated by the standardized difference between the two groups, before and after matching. Standardized differences in patient characteristics, which were below 0.1 across the groups, demonstrate substantial improvement in the balance of covariates [15]. Risk factors for mortality were analyzed with Cox proportional hazards models. First, all covariates for which the *P*-value was < 0.1 in the univariate analysis were included in a Cox proportional hazard model. Second, we considered further adjustment for important covariates that might have significant effects on mortality. Third, the dataset included highly related variables such as pulmonary function tests, we choose only representative variable. We evaluated the effect of tiotropium use with a fixed set of covariates based on clinical expertise, and adjusted with significant variables in univariate analysis. Kaplan-Meier survival analysis, log-rank test and Prentice-Wilcoxon tests were used to evaluate differences in mortality. All statistical analyses were performed using R software 3.1.2 version (R Foundation for Statistical Computing, Vienna, Austria), SPSS 22.0 software (IBM Corporation, Armonk, NY, USA) and SAS Enterprise Guide software (version 6.1, SAS Institute, Inc., Cary, NC, USA).

## Results

### Baseline characteristics of the study population and propensity score matching

Among 963 patients with TDL, 193 patients (19.9%) received tiotropium inhaler treatment for more than 360 days. Patients in the tiotropium group appeared to have worse baseline characteristics than did those in the non-tiotropium group (Table 1). Patients in the tiotropium group were older, likelier to be ever-smokers, had higher mMRC dyspnea scores, and higher proportions of concomitant asthma, COPD, and ICS/LABA usage than the non-tiotropium group. Patients in the tiotropium group also had lower pulmonary function, higher X-ray severity scores, and more long-term oxygen therapy use than patients in the non-tiotropium group.

**Table 1:** Baseline characteristics of patients in tiotropium and non-tiotropium groups

	Tiotropium group	Non-tiotropium group	*P* value	SDM
Patients number	193	770		
Age (years)	63.1 ± 10.3	60.8 ± 13.4	0.011	0.190
Male sex	142 (73.6)	513 (66.6)	0.064	0.153
Body mass index, kg/m^2^	21.3 ± 3.6	21.3 ± 3.7	0.871	0.013
Ever-smokers	124 (64.2)	433 (56.2)	0.044	0.164
mMRC dyspnea scale			< 0.001	0.821
- 0	27 (14.0)	378 (49.1)		
- 1	76 (39.4)	196 (25.5)		
- 2	48 (24.9)	97 (12.6)		
- 3	26 (13.5)	63 (8.2)		
- 4	16 (8.3)	36 (4.7)		
Charlson Comorbidity Index	2.1 ± 1.6	2.1 ± 2.2	0.785	0.020
Concomitant asthma	31 (16.1)	45 (5.8)	< 0.001	0.332
Concomitant COPD	126 (65.3)	162 (21.0)	< 0.001	0.998
ICS/LABA usage^a^	96 (49.7)	29 (3.8)	< 0.001	1.215
Pulmonary function tests				
FEV_1_, % predicted	40.4 ± 14.3	62.7 ± 22.9	< 0.001	1.166
FVC, % predicted	60.3 ± 18.0	69.0 ± 21.0	< 0.001	0.445
FEV_1_/FVC ratio, %	51.7 ± 16.6	69.2 ± 16.9	< 0.001	1.045
DLco, % predicted^b^	50.9 ± 21.4	61.0 ± 20.3	< 0.001	0.481
X-ray severity (0 to 6)	3.5 ± 1.3	2.8 ± 1.4	< 0.001	0.485
Long-term oxygen therapy	35 (18.1)	39 (5.1)	< 0.001	0.417

Data are presented as means ± standard deviation or number of patients (%), unless otherwise indicated

*Abbreviations: mMRC* modified Medical Research Council, *COPD* chronic obstructive pulmonary disease, *ICS/LABA* inhaled corticosteroid/long-acting beta-2 agonist, *FEV*_*1*_ forced expiratory volume in 1 s, *FVC* forced vital capacity, *DLco* diffusing capacity for carbon monoxide

^a^Among 125 patients who received ICS/LABA, 70.6% of patients received fluticasone/salmeterol, and 29.4% of patients received budesonide/formoterol

^b^Available in 343 patients in non-tiotropium group and 79 patients in tiotropium group

After propensity score matching, 105 patients were selected from each group. Baseline characteristics were not different between the tiotropium and non-tiotropium groups after propensity score matching (Table 2). Probability distribution of the tiotropium and non-tiotropium groups was shown in Additional file 1.

**Table 2:** Baseline characteristics of patients in tiotropium and non-tiotropium groups after propensity score matching

	Tiotropium group	Non-tiotropium group	*P* value
Patients number	105	105	
Age (years)	63.9 ± 9.7	64.4 ± 9.6	0.715
Male sex	79 (75.2)	76 (72.4)	0.638
Body mass index, kg/m^2^	21.2 ± 3.5	21.2 ± 3.6	0.929
Ever-smokers	67 (63.8)	71 (67.6)	0.561
mMRC dyspnea scale			0.980
- 0	16 (15.2)	17 (16.2)	
- 1	41 (39.0)	40 (38.1)	
- 2	29 (27.6)	30 (28.6)	
- 3	12 (11.4)	13 (12.4)	
- 4	7 (6.7)	5 (4.8)	
Charlson Comorbidity Index	2.2 ± 1.8	2.3 ± 1.8	0.566
Concomitant asthma	14 (13.3)	15 (14.3)	0.842
Concomitant COPD	63 (60.0)	61 (58.1)	0.779
ICS/LABA usage	18 (17.1)	18 (17.1)	> 0.999
Pulmonary function tests			
FEV_1_, % predicted	45.0 ± 15.0	45.3 ± 16.5	0.903
FVC, % predicted	62.0 ± 17.6	62.0 ± 18.8	0.976
FEV_1_/FVC ratio, %	56.0 ± 17.1	55.6 ± 16.4	0.863
DLco, % predicted	52.5 ± 20.9	54.1 ± 14.2	0.688
X-ray severity score (0 to 6)	3.4 ± 1.2	3.3 ± 1.3	0.786
Long-term oxygen therapy	10 (9.5)	13 (12.4)	0.507

Data are presented as means ± standard deviations or as number of patients (%), unless otherwise indicated. *Abbreviations: SDM* standardized difference of means, *mMRC* modified Medical Research Council, *COPD* chronic obstructive pulmonary disease, *ICS/LABA* inhaled corticosteroid/long-acting beta-2 agonist, *FEV*_*1*_ forced expiratory volume in 1 s, *FVC* forced vital capacity, *DLco* diffusing capacity for carbon monoxide

In addition, we classified patients according to airflow limitation (FEV_1_/FVC ratio < 70) at baseline. Baseline characteristics of patients in tiotropium and non-tiotropium groups among patients without airflow limitation was shown in Additional file 2 and data after propensity score matching was shown in Additional file 3. Baseline characteristics of patients in tiotropium and non-tiotropium groups among patients with airflow limitation was shown in Additional file 4 and data after propensity score matching was shown in Additional file 5.

### Comparison of survival between tiotropium and non-tiotropium groups

**Fig. 2:**
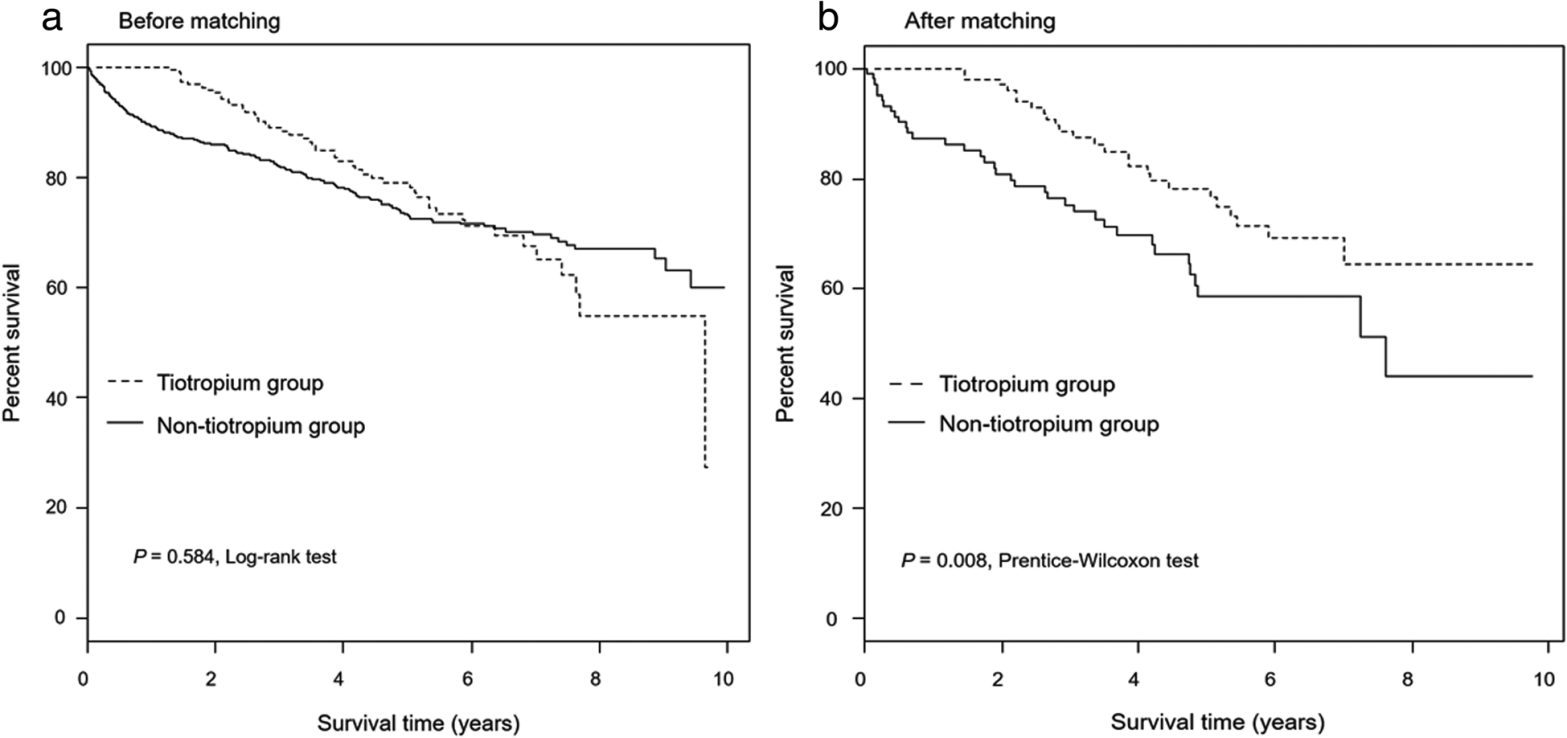
Kaplan-Meier survival curves of tiotropium and non-tiotropium groups. **a** before propensity score matching; (**b**) after propensity score matching

Two-hundred and forty (24.9%) patients died during the follow-up period. Before propensity score matching, there was no difference in survival between tiotropium and non-tiotropium group (median survival: 9.65 years vs. not reached, log-rank test *P* = 0.584) among the total subjects (Fig. 2a). However, after propensity score matching, the survival period in the tiotropium group was significantly longer than those of the non-tiotropium group (median survival: not reached vs. 7.61 years, Prentice-Wilcoxon test *P* = 0.008) (Fig. 2b). Survival analysis was additionally performed in subgroup without airflow limitation. In patients without airflow limitation, there was no difference in survival between tiotropium and non-tiotropium group (log-rank test *P* = 0.279) before propensity score matching. However, after propensity score matching, the survival period in the tiotropium group was tended to be longer than those of the non-tiotropium group (median survival: not reached vs. 3.44 years, Prentice-Wilcoxon test *P* = 0.142) (Additional file 6).

### Risk factors for mortality in patients with TDL

In total subjects with TDL, older age, male sex, higher BMI, ever-smokers, higher mMRC dyspnea scale, and CCI, concomitant COPD, lower FEV_1_, higher X-ray severity score, and long-term oxygen therapy use were significant predictors of mortality in the univariate Cox analysis (Table 3). We evaluated the effect of tiotropium use with a fixed set of covariates based on clinical expertise, and adjusted with significant variables in univariate analysis (age, sex, BMI, smoking history, mMRC dyspnea score, Charlson Comorbidity Index, concomitant COPD diagnosis, FEV_1_, X-ray severity score, and home oxygen usage). Tiotropium inhaler usage was associated with lower risk of mortality in the multivariate analysis (HR, 0.560; 95% CI, 0.380–0.824; *P* = 0.003) and in the propensity score matched analysis (HR, 0.528; 95% CI, 0.316–0.880; *P* = 0.014, Table 4). Forest plot of risk factors for mortality in multivariate Cox analysis was shown in Additional file 7.

**Table 3:** Predicting factors for mortality in patients with tuberculous destroyed lung assessed by univariate cox analysis

	Hazard ratio	95% CI	*p* value
Age, years	1.053	1.040–1.066	< 0.001
Male sex compared to female	3.215	2.255–4.583	< 0.001
Body mass index	0.859	0.827–0.893	< 0.001
Ever-smoker	2.075	1.569–2.746	< 0.001
mMRC dyspnea scale			< 0.001
0 (reference)	1.000		
1	1.497	1.074–2.085	0.017
2	1.742	1.177–2.578	0.006
3	3.139	2.127–4.633	< 0.001
4	3.646	2.235–5.947	< 0.001
Charlson Comorbidity Index	1.234	1.180–1.290	< 0.001
Concomitant asthma	0.980	0.613–1.566	0.932
Concomitant COPD	1.738	1.049–1.784	0.021
ICS/LABA usage	0.849	0.582–1.238	0.394
FEV_1_, % predicted	0.981	0.975–0.987	< 0.001
FVC, % predicted	0.971	0.965–0.978	<.001
FEV_1_/FVC ratio, %	0.996	0.989–1.004	0.316
DLco, % predicted	0.963	0.953–0.973	<.001
X-ray severity (0 to 6)	1.618	1.471–1.780	< 0.001
Long-term oxygen therapy	2.036	1.380–3.004	< 0.001

*Abbreviations: mMRC* modified Medical Research Council, *COPD* chronic obstructive pulmonary disease, *LAMA* long-acting muscarinic antagonist, *ICS/LABA* inhaled corticosteroid/long-acting beta-2 agonist, *FEV1* forced expiratory volume in 1 s, *FVC* forced vital capacity, *DLco* diffusing capacity for carbon monoxide

**Table 4:** Effect of tiotropium inhaler use on mortality in patients with tuberculous destroyed lung

	Hazard ratio	95% CI	*p* value
Univariate analysis	0.916	0.669–1.254	0.585
Multivariate analysis^a^	0.560	0.380–0.824	0.003
Propensity score matched analysis	0.528	0.316–0.880	0.014

^a^Adjusted for age, sex, BMI, smoking history, mMRC dyspnea score, Charlson Comorbidity Index, concomitant chronic obstructive pulmonary disease (COPD) diagnosis, pulmonary function test (FEV_1_), X-ray severity score, and home oxygen usage

### Comparison of outpatient utilization between tiotropium and non-tiotropium groups

Comparisons of outpatient utilization between the tiotropium and non-tiotropium groups are shown in Table 5. The tiotropium group had more outpatient visits compared to non-tiotropium group in both unmatched and matched comparisons. Additionally, the cost of outpatient visits was higher in the tiotropium group than the non-tiotropium group, even after propensity score matching.

**Table 5:** Comparison of outpatient utilization between tiotropium and non-tiotropium groups

	Initial cohort			Matched cohort		
	Tiotropium group	Non-tiotropium group	*P* value	Tiotropium group	Non-tiotropium group	*P*value
Number of patients	193	770		105	105	
Number of outpatient visits	13.3 ± 15.7	4.8 ± 6.7	< 0.001	15.1 ± 20.4	7.8 ± 9.2	0.001
Medical cost for outpatient visit (1000 KRW)	359 ± 936	229 ± 368	< 0.001	672 ± 1086	365 ± 563	0.011

Data are presented as means ± standard deviations

KRW, Korean won, the currency of South Korea

## Discussion

In this study, tiotropium inhaler use might reduce mortality in patients with TDL. Although, the tiotropium group appeared to have worse baseline characteristics compared to the non-tiotropium group, patients in the tiotropium group had better survival than patients in the non-tiotropium group after propensity score matching to adjust for unbalanced baseline characteristics. Although a few studies have examined the lung function of patients with TDL who use inhaler therapy, no study to our knowledge, has investigated the role inhaler therapy may play on mortality outcomes of such patients.

We found that tiotropium inhaler use was independently associated with a favorable prognosis after multivariate analysis. In addition, the survival of patients the tiotropium group was significantly better than those in the non-tiotropium group after propensity score matching. The precise mechanism for this result is unclear, there are some possible explanation. Although there is controversy, a few studies reported that tiotropium might improve survival in patients with COPD [16, 17]. Indeed, our group reported that tiotropium inhaler treatment might reduce mortality in patients with TDL with airflow limitation (FEV1/FVC ratio <  0.70) [18]. In current study, after propensity score matching, approximately 60% of patients with TDL in both the tiotropium and non-tiotropium groups had concomitant COPD, which might have contributed to our results. In our current study, among patients without airflow limitation, the survival period in the tiotropium group was tended to be longer than those of the non-tiotropium group after propensity score matching (median survival: not reached vs. 3.44 years, Prentice-Wilcoxon test *P* = 0.142) (Additional file 6). However, these results might be cautiously interpreted because of small number of patients (8 patients in each groups). Further studies will be needed to confirm this result.

While tiotropium usage was associated with improved survival after propensity score matching, the number of outpatient visits and medical costs remained similar between groups before and after matching. This finding suggests that the tiotropium group visited medical institution in relatively early and mild condition compared to non-tiotropium group. In addition, there is a possibility that tiotropium group had more patients who received active treatment compared to non-tiotropium group in our study. Unfortunately, we did not have a large enough sample of propensity score matched patients to perform additional analysis to adjust for outpatient utilization. Further studies are needed to determine the difference in mortality between tiotropium and non-tiotropium group, after adjusting for healthcare utilization.

No standard management has been developed for patients with TDL. Although surgical treatment was occasionally performed in certain situations such as multi-drug resistance TB [19, 20], it is not easy to perform and might cause complication including empyema and bronchopleural fistula [21]. Prior TB might cause extensive destruction of lung parenchyma and chronic airflow limitation, similar to patients with COPD [22, 23]. Lee et al. reported that previous TB was independently associated with airflow obstruction (OR, 2.56) after adjustment for sex, age, and smoking history using population-based data from the Korea National Health and Nutrition Examination Survey [24]. Thus, inhaler therapies such as long-acting muscarinic antagonists (LAMA) and ICS/LABA are often used in real practice [7]. Although a few studies reported that inhalers treatment might improve lung function [8, 25], there is limited information the effect of inhaler therapy on mortality in patients with TDL. Our current study suggests that tiotropium inhaler therapy can be an appropriate treatment option for these patients.

In addition to tiotropium usage, older age, male sex, lower BMI, higher CCI, and lower FEV_1_ were independent risk factors for mortality, which are similar in patients with COPD [26–28]. Higher X-ray severity scores were also associated with increased mortality in patients with TDL, agreeing with findings of another study in Korea [29]. On the other hand, Kim et al. reported that severity of chest X-ray was not associated with mortality in patients with TDL receiving mechanical ventilation [3]. However, they enrolled relatively small patients and only patients who were admitted to the intensive care unit which making it difficult to generalize their results.

The greatest strength of this study is the comprehensive capture the follow up data such as health care utilization via using national health insurance claims database. Because we investigated all medical claim data in each patient, including healthcare utilization in other hospital, this method made more accurate data and reflected real-world practice. However, there are several limitations to this study. First, our study had retrospective design and that it was conducted in a single tertiary referral center. Therefore, time sequence and causal relationship might be vague. In addition, although the propensity score match has been done, some mismatch could not be seen in this relatively small number of patients. Furthermore, since many patients who were initially diagnosed and treated tuberculosis in other hospital were referred, it is hard to now TB background and the clinical course of getting obstructive airway after TB in study population. But, the HIRA database covers the entire population of Korea (50 million), all information of medical institution visits (inpatient, outpatient and pharmacy visits) in Korea included the database. However, cause of death, general health status and changes in severity of condition were absent in the HIRA database [30]. Thus, we could not evaluate the effect of infection or acceleration on mortality in patients with TDL. Second, the treatment duration was variable even in the tiotropium group, that is, a medication possession ratio (MPR) was diverse in this group. In addition, it is unclear whether the prescribed tiotropium was actually administered in each patient. However, we defined tiotropium group as only patients who were prescribed the tiotropium inhaler for more than 360 days, which might enable to evaluate long term effect of tiotropium. Also, according to hospital medical record, the median tiotropium prescribe duration was relatively long period (median 1020 days, [interquartile range 600–1480 days]). Among tiotropium group, the MPR during 2 years after TDL diagnosis was over than 0.5 in all patients and most patients were in the range of 0.5 to 0.8 (88.1% of initial cohort and 84.8% of matched cohort), which suggest that most patients used tiotropium relatively well, especially in early phase. Third, information about cause of death was not available even in HIRA database, which might induce potential bias to our results; however, we tried to minimize other missing data, by linkage between hospital data and nationwide medical claim data. Finally, although the number of patients in this study was relatively large in comparison with those in previous studies, it is still too small to perform meaningful comparisons within specific conditions such as without airflow limitation (FEV_1_/forced vital capacity [FVC] ratio ≥ 0.7). Despite of these limitations, our current study investigated the effect of tiotropium on mortality in patients with TDL, which was not previously reported.

## Conclusion

In conclusion, tiotropium inhaler therapy might be associated with reducing all-cause mortality in patients with TDL. Further prospective studies involving larger sample sizes will be needed to validate our results.

## Additional files

Additional file 1: Probability distribution of tiotropium and non-tiotropium groups. (a) before propensity score matching; (b) after propensity score matching. (TIFF 6251 kb)

Additional file 2: Baseline characteristics of patients in tiotropium and non-tiotropium groups among patients without airflow limitation (FEV_1_/FVC ratio ≥ 0.7). (DOCX 16 kb)

Additional file 3: Baseline characteristics of patients in tiotropium and non-tiotropium groups among patients without airflow limitation (FEV_1_/FVC ratio ≥ 0.7) after propensity score matching. (DOCX 15 kb)

Additional file 4: Baseline characteristics of patients in tiotropium and non-tiotropium groups among patients with airflow limitation (FEV_1_/FVC ratio < 0.7). (DOCX 16 kb)

Additional file 5: Baseline characteristics of patients in tiotropium and non-tiotropium groups among patients without airflow limitation (FEV_1_/FVC ratio < 0.7) after propensity score matching (DOCX 15 kb)

Additional file 6: Kaplan-Meier survival curves of tiotropium and non-tiotropium groups among patient without airflow limitation. (a) before propensity score matching; (b) after propensity score matching. (TIFF 101 kb)

Additional file 7: Forest plot of risk factors for mortality in patients with tuberculous destroyed lung (results from multivariate analysis). (TIFF 4774 kb)

## Abbreviations

BMI: Body mass index
CCI: Charlson Comorbidity Index
DLco: Diffusing capacity for carbon monoxide
FEV1: Forced expiratory volume in 1 s
FVC: Forced vital capacity
HIRA: Health Insurance Review and Assessment Service
ICS/LABA: Inhaled corticosteroid/long-acting beta-2 agonist
IRR: Incidence rate ratio
LAMA: Long acting muscarinic antagonists
mMRC: Modified Medical Research Council
OR: Odds ratio
TB: Tuberculosis
TDL: Tuberculous destroyed lung
