# Supplementary figures and images for: Effect of tiotropium inhaler use on mortality in patients with tuberculous destroyed lung: based on linkage between hospital and nationwide health insurance claims data in South Korea
Source: Respir Res. 2019 May 6;20:85. doi: 10.1186/s12931-019-1055-5 (PMC6503445; doi:10.1186/s12931-019-1055-5)

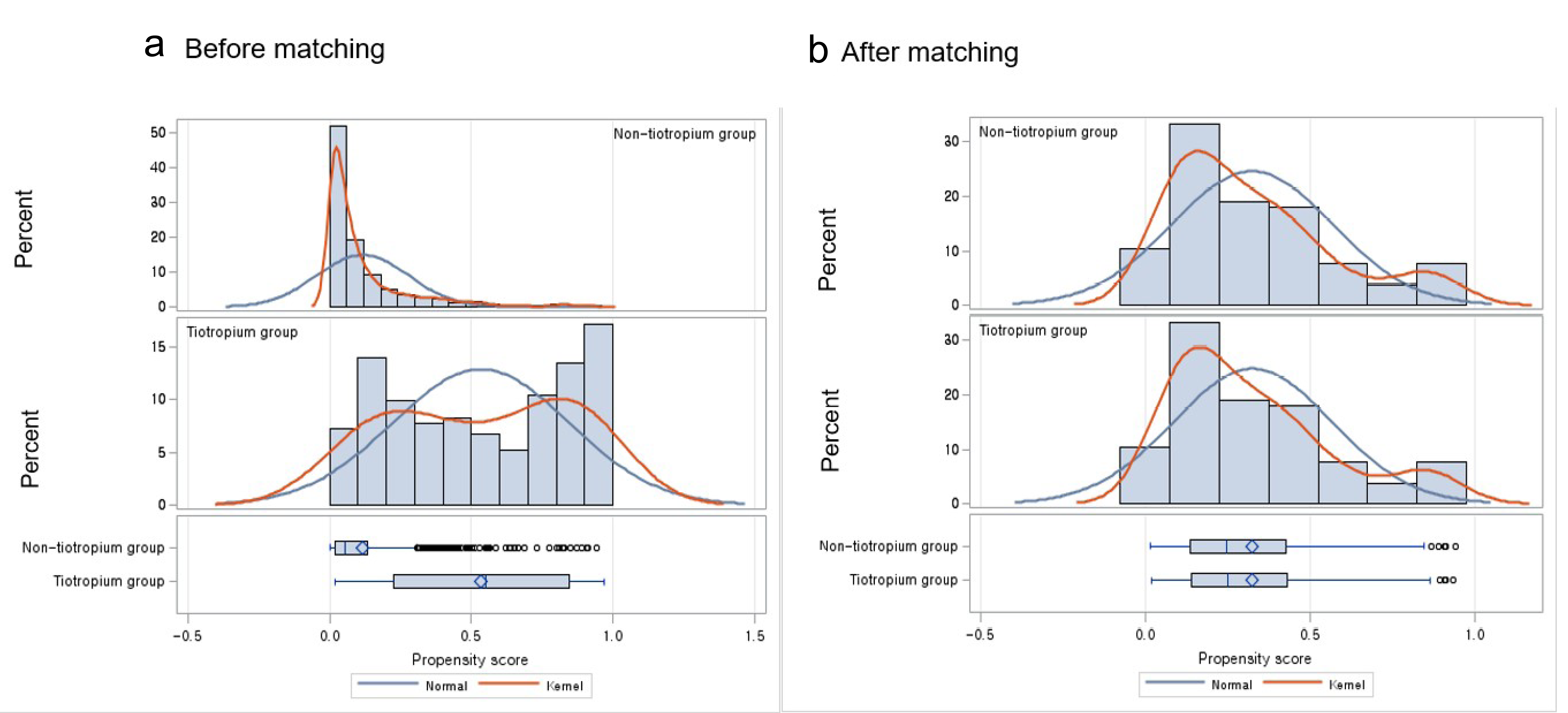

Supplement: Supplementary file 1 — Probability distribution of tiotropium and non-tiotropium groups. (a) before propensity score matching; (b) after propensity score matching. (TIFF 6251 kb) [file 12931_2019_1055_MOESM1_ESM.tiff]

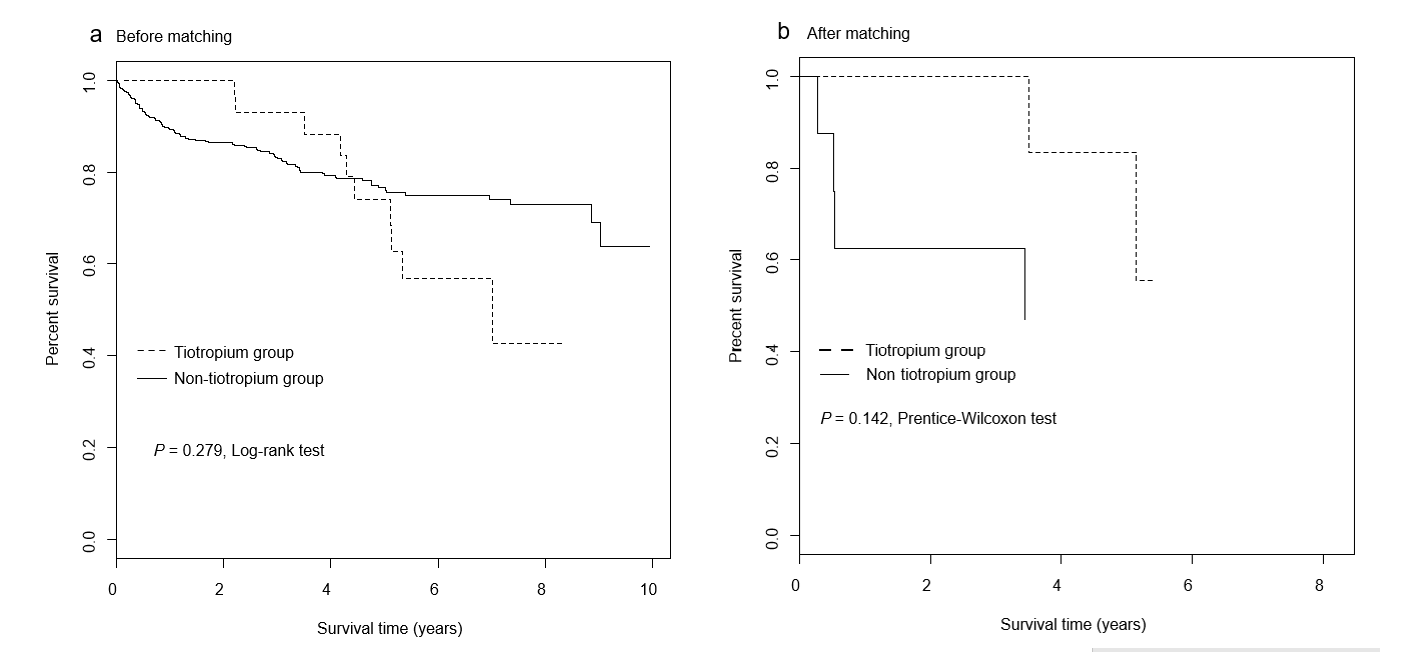

Supplement: Supplementary file 6 — Kaplan-Meier survival curves of tiotropium and non-tiotropium groups among patient without airflow limitation. (a) before propensity score matching; (b) after propensity score matching. (TIFF 101 kb) [file 12931_2019_1055_MOESM6_ESM.tiff]

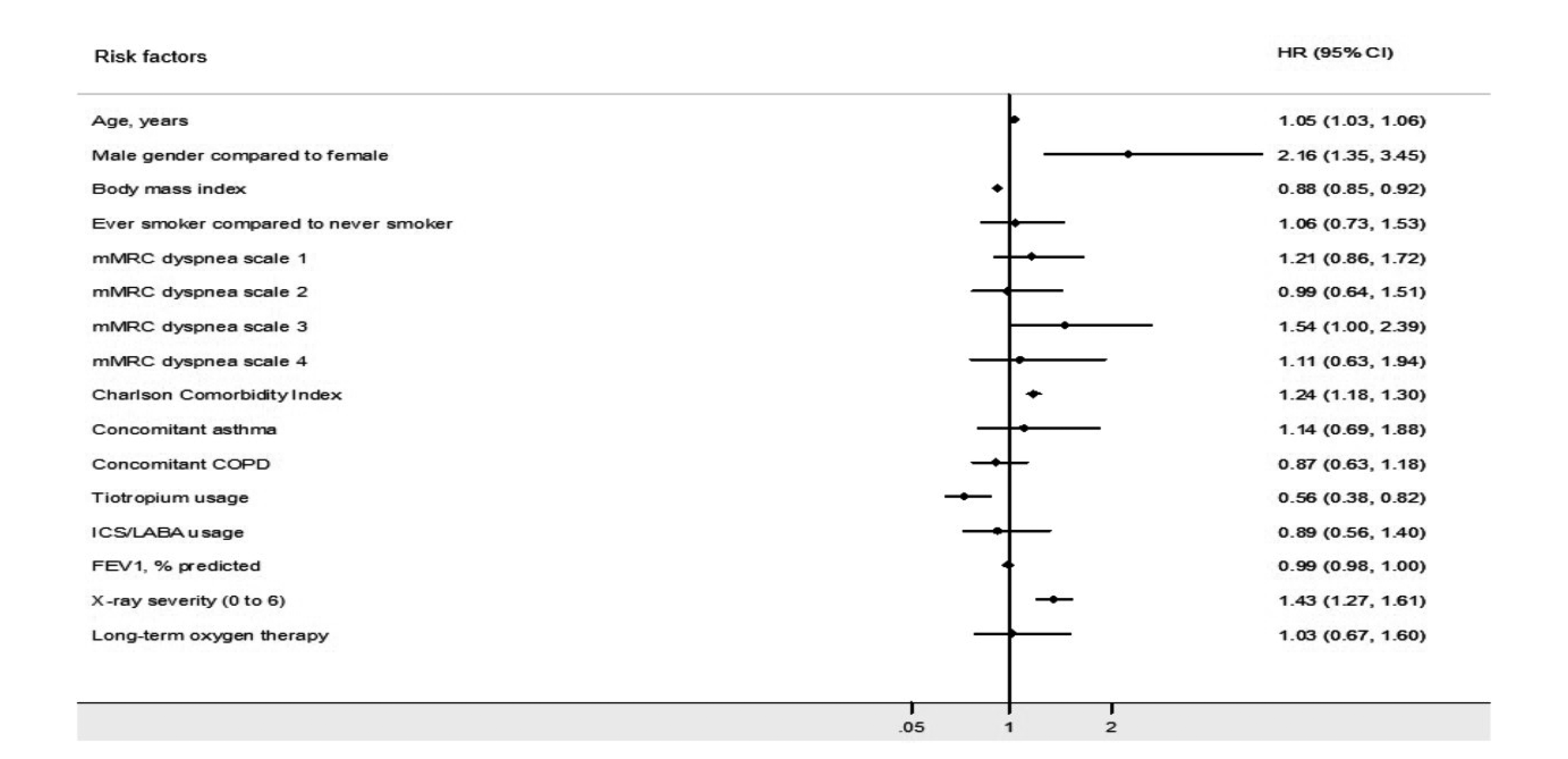

Supplement: Supplementary file 7 — Forest plot of risk factors for mortality in patients with tuberculous destroyed lung (results from multivariate analysis). (TIFF 4774 kb) [file 12931_2019_1055_MOESM7_ESM.tiff]
