# Supplementary material for: Effect of tiotropium inhaler use on mortality in patients with tuberculous destroyed lung: based on linkage between hospital and nationwide health insurance claims data in South Korea
Source: Respir Res. 2019 May 6;20:85. doi: 10.1186/s12931-019-1055-5 (PMC6503445; doi:10.1186/s12931-019-1055-5)
Supplement: Supplementary file 2 — Baseline characteristics of patients in tiotropium and non-tiotropium groups among patients without airflow limitation (FEV1/FVC ratio ≥ 0.7). (DOCX 16 kb) [file 12931_2019_1055_MOESM2_ESM.docx]

**Additional file 2**. Baseline characteristics of patients in tiotropium and non-tiotropium groups among patients without airflow limitation (FEV_1_/FVC ratio ≥ 0.7).

|  | Tiotropium group | Non-tiotropium group | *P* value | SDM |
| --- | --- | --- | --- | --- |
| Patients number | 29 | 401 |  |  |
| Age (years) | 60.0 ± 11.3 | 57.9 ± 14.7 | 0.452 | 0.160 |
| Male sex | 18 (62.1) | 252 (62.8) | 0.934 | -0.016 |
| Body mass index, kg/m^2^ | 20.4 ± 4.4 | 20.7 ± 3.8 | 0.643 | -0.084 |
| Ever-smokers | 13 (44.8) | 196 (48.9) | 0.673 | 0.081 |
| mMRC dyspnea scale |  |  | < 0.001 | 1.066 |
| - 0 | 4 (13.8) | 234 (58.4) |  |  |
| - 1 | 10 (34.5) | 84 (21.0) |  |  |
| - 2 | 7 (24.1) | 43 (10.7) |  |  |
| - 3 | 5 (17.2) | 24 (6.0) |  |  |
| - 4 | 3 (10.3) | 16 (4.0) |  |  |
| Charlson Comorbidity Index | 1.9 ± 1.6 | 1.9 ± 2.1 | 0.982 | 0.005 |
| Concomitant asthma | 3 (10.3) | 11 (2.7) | 0.061 | 0.311 |
| ICS/LABA usage | 11 (37.9) | 6 (1.5) | < 0.001 | 1.030 |
| Pulmonary function tests |  |  |  |  |
| FEV_1_, % predicted | 46.4 ± 18.4 | 71.5 ± 22.0 | < 0.001 | -1.236 |
| FVC, % predicted | 44.3 ± 16.5 | 67.9 ± 22.6 | < 0.001 | 1.193 |
| FEV_1_/FVC ratio, % | 80.0 ± 9.6 | 82.3 ± 9.1 | 0.185 | 0.246 |
| DLco, % predicted | 46.4 ± 19.7 | 61.7 ± 21.2 | 0.020 | 0.748 |
| X-ray severity (0 to 6) | 4.0 ± 1.2 | 2.7 ± 1.5 | < 0.001 | 0.966 |
| Long-term oxygen therapy | 10 (34.5) | 20 (5.0) | < 0.001 | 0.798 |

Data are presented as means ± standard deviation or number of patients (%), unless otherwise indicated.

Abbreviations: SDM, standardized difference of means; mMRC, modified Medical Research Council; ICS/LABA, inhaled corticosteroid/long-acting beta-2 agonist; FEV_1_, forced expiratory volume in 1 second; FVC, forced vital capacity; DLco, diffusing capacity for carbon monoxide.
