# Supplementary material for: Effect of tiotropium inhaler use on mortality in patients with tuberculous destroyed lung: based on linkage between hospital and nationwide health insurance claims data in South Korea
Source: Respir Res. 2019 May 6;20:85. doi: 10.1186/s12931-019-1055-5 (PMC6503445; doi:10.1186/s12931-019-1055-5)
Supplement: Supplementary file 3 — Baseline characteristics of patients in tiotropium and non-tiotropium groups among patients without airflow limitation (FEV1/FVC ratio ≥ 0.7) after propensity score matching. (DOCX 15 kb) [file 12931_2019_1055_MOESM3_ESM.docx]

**Additional file 3**. Baseline characteristics of patients in tiotropium and non-tiotropium groups among patients without airflow limitation (FEV_1_/FVC ratio ≥ 0.7) after propensity score matching

|  | Tiotropium group | Non-tiotropium group | SDM |
| --- | --- | --- | --- |
| Patients number | 8 | 8 |  |
| Age (years) | 64.3 ± 12.3 | 63.9 ± 14.3 | 0.028 |
| Male sex | 6 (75.0) | 5 (62.5) | 0.272 |
| Body mass index, kg/m^2^ | 20.5 ± 4.3 | 18.6 ± 4.7 | 0.435 |
| Ever-smokers | 5 (62.5) | 5 (62.5) | 0.000 |
| mMRC dyspnea scale |  |  | 0.727 |
| - 0 | 3 (37.5) | 2 (25.0) |  |
| - 1 | 1 (12.5) | 2 (25.0) |  |
| - 2 | 2 (25.0) | 2 (25.0) |  |
| - 3 | 2 (25.0) | 1 (12.5) |  |
| - 4 | 0 | 1 (12.5) |  |
| Charlson Comorbidity Index | 1.4 ± 0.5 | 1.5 ± 1.1 | -0.149 |
| Concomitant asthma | 0 | 1 (12.5) | -0.535 |
| ICS/LABA usage | 0 | 1 (12.5) | -0.535 |
| Pulmonary function tests |  |  |  |
| FEV_1_, % predicted | 60.6 ± 14.9 | 59.9 ± 20.8 | 0.041 |
| FVC, % predicted | 55.6 ± 14.8 | 53.0 ± 24.6 | 0.128 |
| FEV_1_/FVC ratio, % | 82.3 ± 11.4 | 88.3 ± 10.7 | 0.543 |
| DLco, % predicted | 57.3 ± 27.4 | 34.6 ± 5.1 | 1.150 |
| X-ray severity score (0 to 6) | 3.4 ± 1.3 | 3.3 ± 1.9 | 0.077 |
| Long-term oxygen therapy | 1 (12.5) | 1 (12.5) | 0.000 |

Data are presented as means ± standard deviations or as number of patients (%), unless otherwise indicated.

Abbreviations: SDM, standardized difference of means; mMRC, modified Medical Research Council; ICS/LABA, inhaled corticosteroid/long-acting beta-2 agonist; FEV_1_, forced expiratory volume in 1 second; FVC, forced vital capacity; DLco, diffusing capacity for carbon monoxide.
