# Supplementary material for: Effect of tiotropium inhaler use on mortality in patients with tuberculous destroyed lung: based on linkage between hospital and nationwide health insurance claims data in South Korea
Source: Respir Res. 2019 May 6;20:85. doi: 10.1186/s12931-019-1055-5 (PMC6503445; doi:10.1186/s12931-019-1055-5)
Supplement: Supplementary file 4 — Baseline characteristics of patients in tiotropium and non-tiotropium groups among patients with airflow limitation (FEV1/FVC ratio < 0.7). (DOCX 16 kb) [file 12931_2019_1055_MOESM4_ESM.docx]

**Additional file 4**. Baseline characteristics of patients in tiotropium and non-tiotropium groups among patients with airflow limitation (FEV_1_/FVC ratio < 0.7).

|  | Tiotropium group | Non-tiotropium group | *P* value | SDM |
| --- | --- | --- | --- | --- |
| Patients number | 164 | 369 |  |  |
| Age (years) | 63.6 ± 10.1 | 63.9 ± 11.0 | 0.743 | -0.031 |
| Male sex | 124 (75.6) | 261 (70.7) | 0.246 | 0.110 |
| Body mass index, kg/m^2^ | 21.5 ± 3.4 | 21.9 ± 3.4 | 0.244 | -0.109 |
| Ever-smokers | 108 (65.9) | 222 (61.8) | 0.370 | 0.085 |
| mMRC dyspnea scale |  |  | < 0.001 | 0.601 |
| - 0 | 23 (14.0) | 144 (39.0) |  |  |
| - 1 | 66 (40.2) | 112 (30.4) |  |  |
| - 2 | 41 (25.0) | 54 (14.6) |  |  |
| - 3 | 21 (12.8) | 39 (10.6) |  |  |
| - 4 | 13 (7.9) | 20 (5.4) |  |  |
| Charlson Comorbidity Index | 2.1 ± 1.6 | 2.4 ± 2.3 | 0.083 |  |
| Concomitant asthma | 28 (17.1) | 34 (9.2) | 0.009 | 0.234 |
| ICS/LABA usage | 113 (68.9) | 133 (36.0) | < 0.001 | 1.162 |
| Pulmonary function tests |  |  |  |  |
| FEV_1_, % predicted | 39.4 ± 13.2 | 53.1 ± 19.9 | < 0.001 | 0.811 |
| FVC, % predicted | 63.2 ± 16.7 | 70.2 ± 19.0 | < 0.001 | 0.391 |
| FEV_1_/FVC ratio, % | 46.7 ± 11.9 | 55.0 ± 10.9 | < 0.001 | 0.727 |
| DLco, % predicted | 51.7 ± 21.7 | 60.1 ± 19.2 | 0.004 | 0.410 |
| X-ray severity (0 to 6) | 3.4 ± 1.3 | 3.0 ± 1.3 | 0.001 | 0.338 |
| Long-term oxygen therapy | 25 (15.2) | 19 (5.2) | < 0.001 | 0.335 |

Data are presented as means ± standard deviation or number of patients (%), unless otherwise indicated.

Abbreviations: SDM, standardized difference of means; mMRC, modified Medical Research Council; ICS/LABA, inhaled corticosteroid/long-acting beta-2 agonist; FEV_1_, forced expiratory volume in 1 second; FVC, forced vital capacity; DLco, diffusing capacity for carbon monoxide.
