# Supplementary material for: Effect of tiotropium inhaler use on mortality in patients with tuberculous destroyed lung: based on linkage between hospital and nationwide health insurance claims data in South Korea
Source: Respir Res. 2019 May 6;20:85. doi: 10.1186/s12931-019-1055-5 (PMC6503445; doi:10.1186/s12931-019-1055-5)
Supplement: Supplementary file 5 — Baseline characteristics of patients in tiotropium and non-tiotropium groups among patients without airflow limitation (FEV1/FVC ratio < 0.7) after propensity score matching (DOCX 15 kb) [file 12931_2019_1055_MOESM5_ESM.docx]

**Additional file 5.** Baseline characteristics of patients in tiotropium and non-tiotropium groups among patients without airflow limitation (FEV_1_/FVC ratio < 0.7) after propensity score matching

|  | Tiotropium group | Non-tiotropium group | SDM |
| --- | --- | --- | --- |
| Patients number | 72 | 72 |  |
| Age (years) | 63.7 ± 9.6 | 64.4 ± 8.5 | -0.070 |
| Male sex | 56 (77.8) | 53 (73.6) | 0.097 |
| Body mass index, kg/m^2^ | 21.4 ± 3.3 | 21.8 ± 3.5 | -0.112 |
| Ever-smokers | 49 (68.1) | 48 (66.7) | 0.030 |
| mMRC dyspnea scale |  |  | 0.178 |
| - 0 | 8 (11.1) | 10 (13.9) |  |
| - 1 | 27 (37.5) | 30 (41.7) |  |
| - 2 | 24 (33.3) | 19 (26.4) |  |
| - 3 | 9 (12.5) | 8 (11.1) |  |
| - 4 | 4 (5.6) | 5 (6.9) |  |
| Charlson Comorbidity Index | 2.3 ± 1.7 | 2.5 ± 1.9 | -0.107 |
| Concomitant asthma | 11 (15.3) | 11 (15.3) | < 0.001 |
| ICS/LABA usage | 14 (19.4) | 15 (20.8) | -0.035 |
| Pulmonary function tests |  |  |  |
| FEV_1_, % predicted | 42.2 ± 12.8 | 43.7 ± 16.0 | -0.106 |
| FVC, % predicted | 64.4 ± 15.7 | 65.8 ± 17.8 | 0.083 |
| FEV_1_/FVC ratio, % | 49.4 ± 12.1 | 48.8 ± 10.4 | 0.053 |
| DLco, % predicted | 52.4 ± 20.8 | 56.1 ± 15.2 | 0.203 |
| X-ray severity score (0 to 6) | 3.3 ± 1.2 | 3.2 ± 1.3 | 0.077 |
| Long-term oxygen therapy | 5 (6.9) | 8 (11.1) | -0.146 |

Data are presented as means ± standard deviations or as number of patients (%), unless otherwise indicated.

Abbreviations: SDM, standardized difference of means; mMRC, modified Medical Research Council; ICS/LABA, inhaled corticosteroid/long-acting beta-2 agonist; FEV_1_, forced expiratory volume in 1 second; FVC, forced vital capacity; DLco, diffusing capacity for carbon monoxide.
